# Association between iron status markers and kidney outcome in patients with chronic kidney disease

**DOI:** 10.1038/s41598-023-45580-8

**Published:** 2023-10-25

**Authors:** Hironobu Fujisawa, Masaru Nakayama, Naoki Haruyama, Akiko Fukui, Ryota Yoshitomi, Kazuhiko Tsuruya, Toshiaki Nakano, Takanari Kitazono

**Affiliations:** 1 Division of Nephrology, Department of Internal Medicine, NHO Kyushu Medical Center, 1-8-1 Jigyohama, Chuo-ku, Fukuoka City, 810-8563 Japan; 2 Department of Nephrology, Nara Medical University, Kashihara City, Japan; 3 Department of Medicine and Clinical Science, Graduate School of Medical Sciences, Kyushu University, Fukuoka City, Japan

**Keywords:** Nephrology, Risk factors

## Abstract

Several studies conducted in patients with various stages of chronic kidney disease (CKD) have investigated the association of iron status markers, such as transferrin saturation (TSAT) and serum ferritin, with kidney outcomes. However, the associations were inconsistent and remain strongly debated. Therefore, we aimed to investigate whether TSAT and serum ferritin levels were associated with kidney outcome in such a population. In this study, 890 patients who were admitted for the evaluation of and education for CKD were prospectively followed. Primary kidney outcome was a composite of doubling of serum creatinine, end-stage kidney disease, or death due to kidney failure. Participants were divided into quartiles (Q1–Q4) according to TSAT or serum ferritin levels. During a median follow-up period of 2.8 years, kidney events occurred in 358 patients. In the multivariable Cox analyses, compared with Q3 of TSAT, the hazard ratios (95% confidence intervals) for Q1, Q2, and Q4 were 1.20 (0.87, 1.66), 1.38 (1.01, 1.87), and 1.14 (0.82, 1.59), respectively. Compared with Q2 of serum ferritin, lower and higher quartiles had a significantly increased risk for kidney outcome; hazard ratios (95% confidence intervals) for Q1, Q3, and Q4 were 1.64 (1.18, 2.27), 1.71 (1.24, 2.37), and 1.52 (1.10, 2.10), respectively. A Fine-Gray model with death before kidney events as a competing risk showed results similar to the above. In CKD, lower and higher ferritin levels were independent risk factors for kidney disease progression.

## Introduction

Iron is vital for several biological processes, including heme synthesis, mitochondrial respiration, DNA synthesis, iron-dependent catalytic reaction oxygen transport, and energy production. Inadequate or excessive bodily iron is associated with various pathological consequences. Iron excess can play key roles in metal-catalyzed oxidative reactions to generate reactive oxygen species, leading to oxidative damage of many cellular components^1–3^. Conversely, iron deficiency (ID) causes adverse clinical consequences through either hematopoietic or nonhematopoietic pathways^4–6^.

In clinical practice, serum ferritin is most commonly used for the evaluation of storage iron, and transferrin saturation (TSAT) is the most commonly applied measure of the iron availability to support erythropoiesis^7^. Serum ferritin is also widely known as an acute-phase reactant and marker of chronic inflammation and is nonspecifically elevated in the setting of inflammatory conditions, including chronic kidney disease (CKD), rheumatoid arthritis and other autoimmune diseases^8,9^. Additionally, TSAT has acute-phase reactivity as transferrin levels are decreased under inflammatory conditions^10^. Therefore, the interpretation of these iron status markers is complicated by concomitant inflammation.

Two types of ID (i.e., absolute and functional ID), assessed by TSAT and serum ferritin levels, are frequently observed in patients with CKD not receiving dialysis. While absolute ID is defined as severely reduced or absent iron stores, functional ID is defined as adequate iron stores but insufficient iron availability for incorporation into erythroid precursors^11–14^. Disordered iron homeostasis, such as absolute or functional ID and iron excess, is reportedly associated with all-cause mortality, heart failure events, and cardiovascular hospitalization in patients with CKD not undergoing dialysis^5,15–17^.

To date, several studies have addressed the issues regarding the associations between iron status markers and kidney outcome in patients with CKD not receiving dialysis. In the Chronic Renal Insufficiency Cohort (CRIC) Study, compared with the iron replete group, participants with mixed ID (iron indices between the absolute and functional ID groups) or high iron had a significant increase in the risk of end-stage kidney disease (ESKD)^15^, whereas another study demonstrated that neither absolute nor functional ID was associated with dialysis initiation in anemic patients with CKD^17^. Furthermore, a few studies investigating the effects of iron status on kidney outcome assessed TSAT and serum ferritin values separately in predialysis CKD. One study reported that higher serum ferritin levels were associated with poor kidney outcome; however, the association between TSAT and kidney outcome was not investigated^18^. Another study demonstrated that higher TSAT levels, but not serum ferritin, were associated with steeper slopes of the estimated glomerular filtration rate (eGFR)^19^. In this context, the associations between iron status markers and kidney outcome are inconsistent and remain strongly debated. Additionally, it remains unclear to what extent TSAT or serum ferritin levels are associated with poor kidney outcome. Therefore, we aimed to investigate whether TSAT or serum ferritin levels are associated with kidney disease progression in patients with CKD not receiving dialysis.

## Results

### Baseline clinical features of patients

In total, 890 patients (male, *n* = 576; female, *n* = 314) with CKD were enrolled in this observational study. The median age of the 890 patients was 69 years (range, 20–94 years). The median eGFR for all participants was 31.3 mL/min/1.73 m^2^ (range, 8.1–138.2 mL/min/1.73 m^2^). Of the 890 patients, 170 (19%), 108 (12%), 190 (21%), 260 (29%), and 162 (18%) were categorized as CKD stage G1–2, G3a, G3b, G4, and G5, respectively. Baseline clinical features of participants according to quartiles of TSAT are presented in Table 1. Participants with lower TSAT levels were older, showed a lower prevalence of male sex, and were more likely to have diabetes mellitus, hypertension and dyslipidemia. The prevalence of prior CVDs increased with lower quartiles of TSAT. Higher C-reactive protein (CRP) and serum phosphorus levels and lower proteinuria, hemoglobin, and eGFR levels were observed in participants with lower TSAT levels. Serum ferritin levels were lower as TSAT levels decreased. The prevalence of anemia increased with lower TSAT levels. The prevalence of participants who received erythropoiesis-stimulating agents was higher with lower TSAT levels. Additionally, TSAT levels inversely correlated with CRP (*r* =  − 0.139,* P* < 0.01) and total iron binding capacity (TIBC) (*r* =  − 0.190, *P* < 0.01).

**Table 1:** Baseline clinical characteristics of patients according to quartiles of TSAT levels.

Variables		TSAT				*P* for trend
	All	Q1 (*n* = 223)	Q2 (*n* = 222)	Q3 (*n* = 223)	Q4 (*n* = 222)	
	(*n* = 890)	(5.8–25.46%)	(25.5–32.7%)	(32.8–41.25%)	(41.3–80.5%)	
Age (years)	69 (57, 78)	73 (63, 80)	71 (60, 77)	68 (56, 78)	65 (51, 75)	< 0.01
Male, *n* (%)	576 (65)	124 (56)	135 (61)	152 (68)	165 (74)	< 0.01
Diabetes mellitus, *n* (%)	321 (36)	92 (41)	89 (40)	67 (30)	73 (33)	0.02
Hypertension, *n* (%)	721 (81)	196 (88)	185 (83)	175 (78)	165 (74)	< 0.01
Systolic blood pressure (mmHg)	132 (119, 144)	134 (121, 145)	131 (118, 144)	130 (119, 142)	131 (118, 146)	0.36
Diastolic blood pressure (mmHg)	72 (66, 80)	71 (65, 78)	72 (66, 79)	73 (65, 81)	74 (68, 83)	< 0.01
Chronic inflammatory disease, *n* (%)	55 (6)	16 (7)	13 (6)	10 (4)	16 (7)	0.86
CVDs, *n* (%)	279 (31)	92 (41)	70 (32)	59 (26)	58 (26)	< 0.01
Smoking, *n* (%)	470 (53)	116 (52)	114 (51)	125 (56)	115 (52)	0.79
Dyslipidemia, *n* (%)	631 (71)	168 (75)	163 (73)	159 (71)	141 (64)	< 0.01
Body mass index (kg/m^2^)	22.9 (20.4, 25.3)	22.4 (20.3, 24.7)	23.1 (20.7, 25.7)	22.9 (20.2, 25.7)	22.8 (20.4, 25.3)	0.55
C-reactive protein (mg/dL)	0.09 (0.05, 0.18)	0.13 (0.05, 0.28)	0.08 (0.05, 0.18)	0.08 (0.05, 0.15)	0.08 (0.05, 0.15)	< 0.01
Daily proteinuria (g/day)	0.94 (0.25, 2.61)	0.69 (0.17, 2.04)	0.91 (0.24, 2.53)	1.09 (0.35, 2.68)	1.22 (0.32, 3.28)	< 0.01
Hemoglobin (g/dL)	11.2 (9.7, 12.8)	10.5 (9.0, 11.7)	11.0 (9.7, 12.2)	11.4 (9.9, 13.1)	12.4 (10.8, 13.8)	< 0.01
Anemia, *n* (%)	469 (53)	152 (68)	121 (55)	116 (52)	80 (36)	< 0.01
eGFR (mL/min/1.73 m^2^)	31.3 (17.9, 52.1)	26.6 (15.9, 43.2)	26.6 (16.6, 41.7)	31.4 (18.1, 53.9)	39.8 (25.3, 67.4)	< 0.01
Serum albumin (g/dL)	3.5 (3.1, 3.8)	3.5 (3.1, 3.7)	3.5 (3.2, 3.8)	3.5 (3.2, 3.8)	3.4 (2.9, 3.8)	0.29
Serum phosphorus (mg/dL)	3.7 (3.3, 4.1)	3.8 (3.3, 4.2)	3.8 (3.4, 4.2)	3.6 (3.3, 4.0)	3.4 (3.1, 3.9)	< 0.01
Serum iron (mg/dL)	81 (61, 103)	50 (40, 63)	73 (62, 84)	90 (80, 103)	119 (101, 138)	< 0.01
TIBC (mg/dL)	254 (219, 286)	272 (232, 309)	251 (218, 282)	253 (220, 280)	242 (213, 276)	< 0.01
TSAT (%)	32.8 (25.5, 41.3)	20.7 (15.8, 23.1)	29.3 (27.8, 31.1)	36.5 (34.3, 38.6)	48.0 (44.4, 54.8)	< 0.01
Serum ferritin (ng/mL)	139.2 (72.5, 265.5)	80.2 (33.6, 181.3)	153.9 (85.4, 269.8)	151.7 (82.6, 275.2)	196.1 (97.7, 322.7)	< 0.01
Use of immunosuppressants, *n* (%)	54 (6)	13 (6)	16 (7)	12 (5)	13 (6)	0.81
Use of RAAS inhibitors, *n* (%)	537 (60)	141 (63)	140 (63)	129 (58)	127 (57)	0.11
Use of ESA, *n* (%)	83 (9)	33 (15)	25 (11)	12 (5)	13 (6)	< 0.01
Use of folic acid or vitamin B12, *n* (%)	51 (6)	13 (6)	15 (7)	12 (5)	11 (5)	0.57
Use of oral iron, *n* (%)	41 (5)	12 (5)	11 (5)	8 (4)	10 (5)	0.52

Values are expressed as number (percent) or median (interquartile range).

*TSAT*, transferrin saturation; *CVDs*, cardiovascular diseases; *eGFR*, estimated glomerular filtration rate; *TIBC*, total iron binding capacity; *RAAS*, renin–angiotensin–aldosterone system; *ESA*, erythropoiesis-stimulating agents.

Table 2 shows the baseline clinical characteristics of participants according to quartiles of serum ferritin. Participants with higher serum ferritin levels had a higher prevalence of male sex, diabetes mellitus, hypertension, smoking, and dyslipidemia. There was no difference in the prevalence of prior CVDs among quartiles of serum ferritin. When serum ferritin levels were higher, higher body mass index (BMI), CRP, and proteinuria levels and lower TIBC and eGFR levels were found. TSAT levels were higher with increasing serum ferritin levels. The prevalence of anemia was not different among quartiles of serum ferritin. The prevalence of participants receiving renin–angiotensin–aldosterone system (RAAS) inhibitors was higher with higher quartiles of serum ferritin. Additionally, a positive association between serum ferritin levels and CRP was found (*r* = 0.101, *P* < 0.01), while serum ferritin levels inversely correlated with TIBC (*r* =  − 0.371, *P* < 0.01).

**Table 2:** Baseline clinical characteristics of patients according to quartiles of serum ferritin levels.

Variables	Serum ferritin				*P* for trend
	Q1 (*n* = 223)	Q2 (*n* = 222)	Q3 (*n* = 223)	Q4 (*n* = 222)	
	(6.4–72.5 ng/mL)	(73.3–139.0 ng/mL)	(139.3–265.5 ng/mL)	(265.6–997.7 ng/mL)	
Age (years)	70 (47, 79)	69 (60, 77)	69 (59, 78)	68 (58, 77)	0.96
Male, *n* (%)	92 (41)	135 (61)	158 (71)	191 (86)	< 0.01
Diabetes mellitus, *n* (%)	68 (30)	79 (36)	88 (39)	86 (39)	0.047
Hypertension, *n* (%)	162 (73)	169 (76)	189 (85)	201 (91)	< 0.01
Systolic blood pressure (mmHg)	129 (117, 141)	132 (118, 145)	131 (119, 144)	134 (122, 147)	< 0.01
Diastolic blood pressure (mmHg)	71 (65, 77)	72 (66, 79)	73 (67, 81)	74 (67, 83)	< 0.01
Chronic inflammatory disease, *n* (%)	16 (7)	18 (8)	11 (5)	10 (5)	0.12
CVDs, *n* (%)	70 (31)	71 (32)	71 (32)	67 (30)	0.79
Smoking, *n* (%)	97 (44)	111 (50)	120 (54)	142 (64)	< 0.01
Dyslipidemia, *n* (%)	136 (61)	166 (75)	164 (74)	165 (74)	< 0.01
Body mass index (kg/m^2^)	21.4 (19.4, 23.9)	22.1 (19.9, 24.6)	23.5 (21.4, 25.9)	23.8 (21.6, 26.5)	< 0.01
C-reactive protein (mg/dL)	0.07 (0.05, 0.17)	0.08 (0.05, 0.16)	0.09 (0.05, 0.18)	0.10 (0.05, 0.20)	< 0.01
Daily proteinuria (g/day)	0.61 (0.19, 1.74)	0.95 (0.29, 2.52)	1.10 (0.21, 3.00)	1.32 (0.36, 3.21)	< 0.01
Hemoglobin (g/dL)	11.0 (9.6, 12.4)	11.2 (10.0, 12.7)	11.3 (9.7, 13.3)	11.4 (9.5, 13.3)	0.06
Anemia, *n* (%)	119 (53)	114 (51)	115 (52)	121 (55)	0.81
eGFR (mL/min/1.73 m^2^)	33.8 (18.0, 63.9)	34.0 (19.5, 53.3)	30.5 (18.5, 50.2)	26.7 (15.5, 43.0)	< 0.01
Serum albumin (g/dL)	3.5 (3.1, 3.7)	3.5 (3.2, 3.8)	3.5 (3.0, 3.8)	3.5 (3.0, 3.8)	0.80
Serum phosphorus (mg/dL)	3.7 (3.3, 4.1)	3.6 (3.2, 4.1)	3.7 (3.2, 4.1)	3.7 (3.2, 4.2)	0.88
Serum iron (mg/dL)	77 (53, 101)	85 (64, 104)	84 (65, 104)	81 (63, 104)	0.02
TIBC (mg/dL)	283 (247, 317)	256 (221, 276)	246 (209, 276)	232 (199, 268)	< 0.01
TSAT (%)	26.5 (19.4, 35.9)	33.1 (26.4, 41.0)	33.7 (27.7, 42.3)	35.5 (28.8, 43.8)	< 0.01
Serum ferritin (ng/mL)	38.2 (25.3, 56.7)	102.9 (87.6, 119.4)	196.3 (166.4, 225.7)	372.3 (306.7, 459.1)	< 0.01
Use of immunosuppressants, *n* (%)	19 (9)	16 (7)	11 (5)	8 (4)	0.02
Use of RAAS inhibitors, *n* (%)	122 (55)	124 (56)	136 (61)	155 (70)	< 0.01
Use of ESA, *n* (%)	21 (9)	20 (9)	16 (7)	26 (12)	0.56
Use of folic acid or vitamin B12, *n* (%)	14 (6)	14 (6)	14 (6)	9 (4)	0.34
Use of oral iron, *n* (%)	13 (6)	6 (3)	9 (4)	13 (6)	0.83

Values are expressed as number (percent) or median (interquartile range).

*CVDs*, cardiovascular diseases; *eGFR*, estimated glomerular filtration rate; *TIBC*, total iron binding capacity; *TSAT*, transferrin saturation; *RAAS*, renin–angiotensin–aldosterone system; *ESA*, erythropoiesis-stimulating agents.

### Associations between iron status markers and kidney outcome

**Figure 1:**
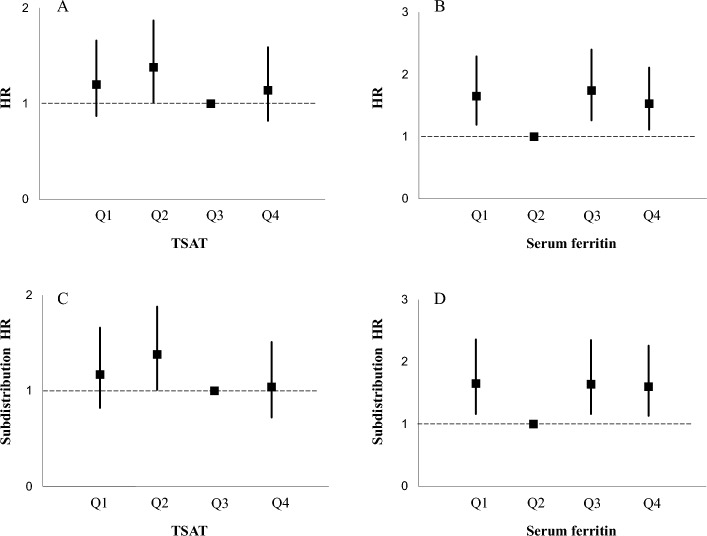
The adjusted HRs (*squares*) and 95% CIs (*vertical lines*) for kidney outcome according to quartiles of TSAT (**A** = Cox model; **C** = Fine-Gray model) and serum ferritin (**B** = Cox model; **D** = Fine-Gray model). Adjusted for the same covariates as model 5 in Table 3. HR, hazard ratio; CI, confidence interval; TSAT, transferrin saturation.

The median follow-up duration in all patients was 2.8 years (range, 0.1–13.4 years). During this period, there were 358 kidney events. Seventy-five participants were lost to follow-up after being followed up for at least 6 months. Table 3 presents the hazard ratios (HRs) and subdistribution HRs for kidney outcome of TSAT and serum ferritin levels. The unadjusted Cox model demonstrated that participants with Q1, Q2, or Q4 of TSAT did not display a significant association with kidney outcome, compared with Q3 of TSAT. In the fully adjusted Cox model (model 5), compared with Q3 of TSAT, participants with Q2 of TSAT had a significant increase in the HRs for kidney outcome, whereas participants with Q1 or Q4 did not display a significant association with kidney outcome. On the other hand, in the unadjusted Cox model, compared with Q2 of serum ferritin, participants with Q3 or Q4 of serum ferritin had a significant increase in the HRs for kidney outcome, but those with Q1 of serum ferritin did not display a significant association with kidney outcome. Conversely, in the adjusted Cox models (from model 2 to model 5), participants with Q1, Q3, or Q4 of serum ferritin had significantly higher HRs for adverse kidney outcome compared with Q2 of serum ferritin. In addition, when the Fine-Gray model with all-cause death before kidney events (*n* = 73) as a competing risk was performed, the associations between iron status markers and kidney outcome were similar to the above results (Cox models). Figure 1 presents the HRs (95% confidence intervals [CIs]) for kidney outcome according to quartiles of TSAT and serum ferritin.

**Table 3:** Adjusted HRs and SHRs for kidney outcome of TSAT and serum ferritin levels.

No. of events	TSAT						
	Q1		Q2		Q3	Q4	
	101		107		82	68	
	HR (95% CI)	*P*	HR (95% CI)	*P*		HR (95% CI)	*P*
Model 1	1.24 (0.92, 1.66)	0.15	1.33 (0.996, 1.77)	0.05	Reference	0.77 (0.56, 1.06)	0.11
Model 2	1.19 (0.89, 1.60)	0.24	1.26 (0.94, 1.68)	0.12	Reference	0.76 (0.55, 1.05)	0.09
Model 3	1.03 (0.76, 1.38)	0.87	1.26 (0.94, 1.68)	0.13	Reference	0.72 (0.52, 0.996)	0.047
Model 4	1.10 (0.80, 1.50)	0.56	1.33 (0.98, 1.81)	0.06	Reference	1.17 (0.84, 1.63)	0.36
Model 5	1.20 (0.87, 1.66)	0.26	1.38 (1.01, 1.87)	0.04	Reference	1.14 (0.82, 1.59)	0.44

	SHR (95% CI)	*P*	SHR (95% CI)	*P*		SHR (95% CI)	*P*
Model 1	1.18 (0.88, 1.59)	0.26	1.34 (1.01, 1.78)	0.04	Reference	0.80 (0.58, 1.10)	0.17
Model 2	1.18 (0.87, 1.58)	0.29	1.31 (0.98, 1.74)	0.07	Reference	0.80 (0.58, 1.10)	0.17
Model 3	1.02 (0.75, 1.40)	0.89	1.31 (0.98, 1.76)	0.07	Reference	0.76 (0.55, 1.07)	0.12
Model 4	1.05 (0.74, 1.48)	0.78	1.34 (0.99, 1.83)	0.06	Reference	1.09 (0.76, 1.57)	0.64
Model 5	1.17 (0.82, 1.66)	0.40	1.38 (1.01, 1.88)	0.04	Reference	1.04 (0.72, 1.51)	0.82

No. of events	Serum ferritin						
	Q1		Q2	Q3		Q4	
	89		74	94		101	
	HR (95% CI)	*P*		HR (95% CI)	*P*	HR (95% CI)	*P*
Model 1	1.24 (0.91, 1.69)	0.17	Reference	1.47 (1.09, 2.00)	0.01	1.84 (1.36, 2.48)	< 0.01
Model 2	1.41 (1.03, 1.92)	0.03	Reference	1.43 (1.06, 1.94)	0.02	1.66 (1.22, 2.25)	< 0.01
Model 3	1.56 (1.14, 2.13)	< 0.01	Reference	1.72 (1.27, 2.35)	< 0.01	1.96 (1.44, 2.67)	< 0.01
Model 4	1.60 (1.16, 2.21)	< 0.01	Reference	1.73 (1.25, 2.39)	< 0.01	1.56 (1.13, 2.14)	< 0.01
Model 5	1.64 (1.18, 2.27)	< 0.01	Reference	1.71 (1.24, 2.37)	< 0.01	1.52 (1.10, 2.10)	< 0.01

	SHR (95% CI)	*P*		SHR (95% CI)	*P*	SHR (95% CI)	*P*
Model 1	1.25 (0.93, 1.70)	0.14	Reference	1.50 (1.11, 2.04)	< 0.01	1.80 (1.33, 2.43)	< 0.01
Model 2	1.41 (1.04, 1.91)	0.03	Reference	1.49 (1.10, 2.01)	0.01	1.64 (1.21, 2.23)	< 0.01
Model 3	1.58 (1.14, 2.19)	< 0.01	Reference	1.77 (1.28, 2.45)	< 0.01	1.96 (1.40, 2.74)	< 0.01
Model 4	1.62 (1.14, 2.32)	< 0.01	Reference	1.66 (1.17, 2.37)	< 0.01	1.62 (1.15, 2.29)	< 0.01
Model 5	1.65 (1.16, 2.36)	< 0.01	Reference	1.64 (1.15, 2.35)	< 0.01	1.60 (1.13, 2.26)	< 0.01

Model 1: crude.

Model 2: adjusted for age and sex.

Model 3: Model 2 plus adjusted for smoking, diabetes mellitus, dyslipidemia, systolic blood pressure, and body mass index.

Model 4: Model 3 plus adjusted for chronic inflammatory disease, CVDs, use of ESA, use of immunosuppressants, use of RAAS inhibitors, C-reactive protein, daily proteinuria, serum albumin, eGFR, hemoglobin, and serum phosphorus.

Model 5: Model 4 plus adjusted for serum ferritin (for TSAT) or TSAT (for serum ferritin).

*HR*, hazard ratio; *SHR*, subdistribution hazard ratio; *TSAT*, transferrin saturation; *CI*, confidence interval; *CVDs*, cardiovascular diseases; *ESA*, erythropoiesis-stimulating agents; *RAAS*, renin–angiotensin–aldosterone system; *eGFR*, estimated glomerular filtration rate.

**Figure 2:**
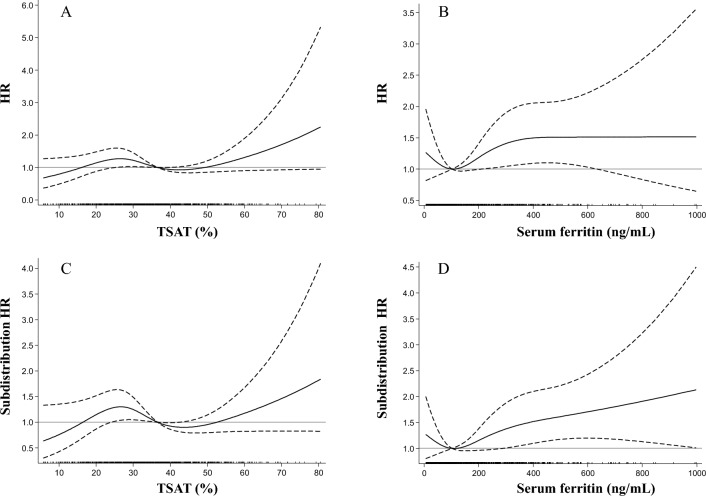
The adjusted HR and subdistribution HR for kidney outcome in TSAT levels (**A** = Cox model; **C** = Fine-Gray model) and in serum ferritin levels (**B** = Cox model; **D** = Fine-Gray model). The solid and dashed lines indicate the HR and the 95% CI, respectively. Adjusted for the same covariates as model 5 in Table 3. HR, hazard ratio; TSAT, transferrin saturation; CI, confidence interval.

A spline analysis of the relationship between serum ferritin levels and the risk of adverse kidney outcome showed that the associations between TSAT levels and the risk of poor kidney outcome did not display formulaic patterns (e.g., linear and J- or U-shaped patterns), whereas the risk of poor kidney outcome increased with both lower and higher serum ferritin levels, showing a J-shaped pattern, as shown in Fig. 2.

### Subgroup analyses regarding associations between serum ferritin and kidney outcome

Table 4 shows the fully adjusted subdistribution HRs for kidney outcome of Q1, Q3, and Q4 of serum ferritin according to subgroups stratified by demographic and clinical features, compared with Q2, using a Fine-Gray model. In male participants and in participants with lower serum albumin levels, the HRs of Q1, Q3, and Q4 significantly increased compared with that of Q2. Participants with lower hemoglobin and eGFR levels showed a significant increase in the risk of poor kidney outcomes in both Q1 and Q4 of the serum ferritin level compared with Q2.

**Table 4:** Adjusted subdistribution hazard ratios for kidney outcome of serum ferritin levels in subgroups.

Groups		No. of patients	No. of events	Serum ferritin							*P* for interaction
				Q1		Q2	Q3		Q4		
				6.4–72.5 ng/mL		73.3–139.0 ng/mL	139.3–265.5 ng/mL		265.6–997.7 ng/mL		
				SHR (95% CI)	*P*		SHR (95% CI)	*P*	SHR (95% CI)	*P*	
Age	Low (< 69.35 years)	445	159	1.55 (0.82, 2.94)	0.18	Reference	1.29 (0.77, 2.18)	0.34	1.21 (0.71, 2.04)	0.48	0.83
	High (≥ 69.35 years)	445	199	1.48 (0.94, 2.33)	0.09	Reference	1.76 (1.11, 2.79)	0.02	1.57 (1.02, 2.40)	0.04	
Sex	Males	576	258	2.26 (1.46, 3.49)	< 0.01	Reference	1.96 (1.29, 2.99)	< 0.01	1.64 (1.09, 2.47)	0.02	0.35
	Females	314	100	1.19 (0.64, 2.23)	0.58	Reference	1.21 (0.61, 2.41)	0.59	1.52 (0.74, 3.10)	0.25	
Body mass index	Low (< 22.87 kg/m^2^)	445	170	1.57 (0.95, 2.61)	0.08	Reference	1.65 (0.94, 2.89)	0.08	2.13 (1.29, 3.50)	< 0.01	0.15
	High (≥ 22.87 kg/m^2^)	445	188	1.80 (1.07, 3.04)	0.03	Reference	1.81 (1.14, 2.87)	0.01	1.22 (0.75, 1.96)	0.43	
Serum albumin	Low (< 3.6 g/dL)	500	241	1.80 (1.13, 2.88)	0.01	Reference	1.87 (1.20, 2.91)	< 0.01	1.81 (1.18, 2.79)	< 0.01	0.29
	High (≥ 3.6 g/dL)	390	117	1.19 (0.67, 2.11)	0.54	Reference	1.07 (0.60, 1.92)	0.83	0.96 (0.53, 1.73)	0.88	
CRP	Low (< 0.10 mg/dL)	473	186	1.38 (0.84, 2.29)	0.20	Reference	1.78 (1.09, 2.92)	0.02	1.68 (1.03, 2.73)	0.04	0.50
	High (≥ 0.10 mg/dL)	417	172	1.80 (1.08, 2.98)	0.02	Reference	1.36 (0.82, 2.24)	0.23	1.48 (0.90, 2.45)	0.12	
Hemoglobin	Low (< 11.3 g/dL)	450	256	1.70 (1.13, 2.55)	0.01	Reference	1.50 (0.998, 2.27)	0.05	1.60 (1.07, 2.39)	0.02	0.76
	High (≥ 11.3 g/dL)	440	102	1.65 (0.84, 3.24)	0.15	Reference	2.45 (1.30, 4.64)	< 0.01	1.64 (0.88, 3.08)	0.12	
eGFR	Low (< 31.3 mL/min/1.73 m^2^)	445	282	1.50 (1.01, 2.23)	0.046	Reference	1.32 (0.88, 1.98)	0.18	1.54 (1.07, 2.23)	0.02	0.55
	High (≥ 31.3 mL/min/1.73 m^2^)	445	76	1.82 (0.75, 4.47)	0.19	Reference	4.39 (1.93, 9.98)	< 0.01	1.68 (0.63, 4.50)	0.30	

Adjusted for age, sex, smoking, diabetes mellitus, dyslipidemia, systolic blood pressure, body mass index, chronic inflammatory disease, CVDs, use of ESA, use of immunosuppressants, use of RAAS inhibitors, C-reactive protein, daily proteinuria, serum albumin, eGFR, hemoglobin, serum phosphorus, and TSAT.

*SHR*, subdistribution hazard ratio; *CI*, confidence interval; *CRP*, C-reactive protein; eGFR, estimated glomerular filtration rate; *CVDs*, cardiovascular diseases; *ESA*, erythropoiesis-stimulating agents; *RAAS*, renin–angiotensin–aldosterone system; *TSAT*, transferrin saturation.

## Discussion

The present study demonstrated a J-shaped association between serum ferritin levels and kidney outcome in patients with CKD. Furthermore, the Fine-Gray model showed a similar association. In Japanese dialysis patients, those with the lowest (< 50 ng/mL) and those with the highest serum ferritin levels (≥ 200 ng/mL) had a significant increase in the risk of all-cause mortality compared with those with serum ferritin of 50–99.9 ng/mL^20^. Another study conducted in Japanese dialysis patients showed a J-shaped association of the high (versus median) ferritin and the lowest ferritin levels with mortality^21^. To date, however, no reports have demonstrated J- or U-shaped associations between serum ferritin levels and poor kidney outcome in CKD. Therefore, the present study was the first report to clarify a J-shaped association.

Bone marrow iron depletion is highly specific for the diagnosis of ID, is unaffected by inflammation, and remains the gold standard for ID diagnosis^22^. A few studies investigated the association between bone marrow iron stores and serum iron status markers in CKD. In patients with CKD, serum ferritin levels < 60 ng/mL were associated with absent or reduced bone marrow iron. Using a critical value of ≤ 60 ng/mL, the diagnostic efficiency of serum ferritin in terms of diagnosing absent or reduced bone marrow iron was high^23^. In another report by Kalantar-Zadeh et al., when bone marrow iron was graded 0 to + 4, ranging from absent to diffuse homogeneous iron staining, serum ferritin concentrations (mean value, 83 ng/mL) in CKD patients with a bone marrow iron score of 0 were significantly lower than in those with a bone marrow iron score of + 2 or greater. It was also noted that there was a significant correlation between serum ferritin and bone marrow iron scores^24^. These findings suggested that the specificity of low serum ferritin is high for absolute ID^25^.

The underlying mechanisms for the association between low serum ferritin and kidney disease progression observed in the present study remains unknown. Deleterious biological consequences of ID are considered multifactorial as follows: impaired erythropoiesis, abnormalities in the immune response, DNA and cell cycle abnormalities, and mitochondrial dysfunction^4–6^. The kidney is a high energy-consuming tissue, and the mitochondria are the major source of cellular ATP^26^. Furthermore, dysfunctional mitochondrial energy production due to ID is associated with kidney impairment^5,6^. In chronic experimental atherosclerotic renovascular disease, loss of mitochondrial inner membrane cardiolipin was reported to contribute to kidney injury and dysfunction^27^. It was also proposed that ID might adversely affect the kidney through oxidative stress as well as mitochondrial dysfunction^6^. As mentioned above, lower serum ferritin is associated with body iron depletion. Given these findings, it is possible that the mitochondrial dysfunction and oxidative stress caused by ID which is probably associated with low serum ferritin are raised as the mechanisms by which the lowest quartile of serum ferritin showed a significant increase in the risk for poor kidney outcome in the present study. However, the effects of iron supplementation for ID on kidney function are scarce^28^. Conversely, in an animal model, iron restriction prevented the development of kidney damage^29^. Therefore, further studies are warranted to investigate whether treatment of ID with iron supplementation improves kidney function in CKD.

In CKD, very few studies have shown a significant association between higher serum ferritin levels and kidney disease progression. Tsai et al. suggested that patients with CKD who displayed serum ferritin levels > 288 ng/mL were more likely to experience adverse kidney outcome compared with those with serum ferritin levels < 132 ng/mL^18^. In the present study, compared with Q2 of serum ferritin (73.3–139.0 ng/mL), Q3 and Q4 (> 139.0 ng/mL) were significantly associated with poor kidney outcome. According to World Health Organization guidelines, in adult, non-healthy individuals, serum ferritin concentration > 500 ng/mL may indicate a risk of iron overload^30^. Serum ferritin values in the range of 200–2,000 ng/mL may be increased due to non-iron associated factors, including elements of malnutrition–inflammation complex syndrome, while extremely high serum ferritin levels of > 2,000 ng/mL are normally indicative of iron overload in patients with CKD^8,31^. In the present study, the number of participants with serum ferritin > 500 ng/mL was very small (*n* = 42; 4.7%); therefore, most participants may have had non-iron related conditions, rather than iron-overload. TIBC is a negative acute-phase reactant, that is, its plasma concentration is suppressed by inflammation^32^. In our cohort, serum ferritin levels positively and inversely correlated with CRP and TIBC, respectively, suggesting that higher serum ferritin levels reflect a greater inflammatory status. Ferritin synthesis is activated by some pro-inflammatory cytokines (e.g., interleukin [IL]-1β and tumor necrosis factor [TNF]- α)^33,34^. Furthermore, these cytokines such as IL-1β^35^and TNF-α^36^ can play an important role in kidney disease progression. Although we did not measure these cytokines (e.g., IL-1β and TNF-α), it was proposed that those cytokines concomitant with elevated serum ferritin might be considered to contribute to kidney disease progression.

In patients with advanced CKD not receiving dialysis, a trend for higher mortality was reportedly observed in patients with a serum ferritin level of > 250 ng/mL, although there was no statistical significance^19^. In a study of the liver iron concentration as measured by magnetic resonance imaging, the cutoff serum ferritin level was 290 ng/mL in patients with severe iron overload undergoing hemodialysis^37^. A national survey conducted by the Japanese Society for Dialysis Therapy in 2012 revealed that the erythropoiesis-stimulating agent resistance index, which reflects hyporesponsiveness, tended to increase at serum ferritin levels of ≥ 300 ng/mL. Additionally, only 10% of Japanese patients undergoing dialysis exhibited serum ferritin levels of ≥ 300 ng/mL. Based on reports including these findings, the Japanese Society for Dialysis Therapy did not recommend iron administration that targets a serum ferritin level of ≥ 300 ng/mL^38^.

By contrast, in a study of Japanese patients undergoing hemodialysis, a serum ferritin level of > 100 ng/mL was associated with a higher mortality risk than a lower serum ferritin level^39^. In another study of Japanese patients undergoing hemodialysis with repeated measurements of serum ferritin, patients who had high serum ferritin levels (> 100 ng/mL) were kept and those who showed high-amplitude fluctuations in serum ferritin during the study period had an increased risk of cerebrovascular and cardiovascular disease, infection, and death^40^. Additionally, another study demonstrated a higher risk for all-cause death in Japanese patients undergoing hemodialysis with higher serum ferritin levels (≥ 200 ng/mL) than the reference range (50.0–99.9 ng/mL)^20^. These studies suggest that even Japanese patients undergoing hemodialysis with serum ferritin levels of < 300 ng/mL may have an increased risk of poor outcomes.

Furthermore, in patients with CKD not undergoing dialysis, studies of the association between the serum ferritin concentration and progression of kidney disease are scarce. To our knowledge, two studies have addressed this association. One study demonstrated no significant association between the serum ferritin concentration and the slope of the eGFR^19^. In another study by Tsai et al.^18^, patients with CKD were divided into tertiles of serum ferritin levels (cutoff of 132 and 288 ng/mL), and multivariable Cox analyses showed that the HRs (95% CIs) for kidney replacement therapy in tertile 2 and tertile 3 were 1.16 (0.99, 1.35) and 1.20 (1.03, 1.40), respectively, compared with tertile 1. In the present study, compared with Q2 of the serum ferritin levels, Q3 (serum ferritin of 139.3–265.5 ng/mL) and Q4 (serum ferritin of 265.6–997.7 ng/mL) were associated with poor kidney outcomes. The range of serum ferritin levels in Q3 in our cohort was similar to that in tertile 2, which tended to be associated with poor kidney outcomes in the study by Tsai et al.^18^. Accordingly, in patients with CKD, even a serum ferritin level of < 300 ng/mL might be associated with kidney disease progression. Additionally, in our study, the HR and subdistribution HR in Q3 were slightly higher than those in Q4 (Table 3 and Fig. 1). This finding may imply that the risk of a poor kidney outcome did not progressively increase with higher serum ferritin levels. Although the existence of some residual confounding may have contributed to this result, the underlying cause remains unclear.

Very few studies have addressed the association between TSAT and kidney outcome in patients with CKD. A previous study conducted in 453 male patients with CKD reported that higher TSAT levels were associated with worsening kidney function^19^. Serum iron levels fluctuate diurnally and can change acutely depending on dietary iron intake. In the present study, blood samples were obtained from all participants early in the morning following an overnight fast; therefore, the measured serum iron levels were unaffected by diurnal variation or dietary intake. Compared with Q3 of TSAT, Q2 displayed a significant increase in the risk of kidney outcome, but neither Q1 nor Q4 was associated with kidney outcome. In this context, it remains unclear to what extent TSAT is associated with kidney outcome. Therefore, larger cohort studies are needed to clarify the association of TSAT levels with kidney outcome.

In the present subgroup analyses, in addition to male participants, participants with a lower serum albumin, hemoglobin, or eGFR level were more likely to have significant associations of both the lowest and highest ferritin levels with poor kidney outcomes. However, there were no significant interactions on kidney outcome between variables (sex, serum albumin, hemoglobin, and eGFR) and quartiles of serum ferritin. The absence of interactions might be explained by the J-shaped association between serum ferritin and kidney outcomes as well as the lack of an intrinsic association of the quartiles of serum ferritin (ordinal variable) with kidney outcomes (data not shown). These results may suggest that patients with malnutrition, anemic conditions, or advanced kidney dysfunction are susceptible to adverse influences of serum ferritin levels on kidney disease progression, although there were no significant interactions in the present study.

The present study had several limitations. First, all study participants were recruited at a single regional hospital. Therefore, the sample was fairly homogeneous, and selection bias was possible. We only recruited consecutive patients who were admitted to our hospital, and these participants were relatively old, with the number of male participants being approximately 1.8-times higher than that of female participants. Second, the present study used single iron status marker measurements, which may not provide sufficient accuracy regarding the predictive values of those factors for kidney outcome. Third, we did not measure serum hepcidin, which is a major iron-regulatory hormone that binds to ferroportin and inhibits iron export from enterocytes, hepatocytes, and macrophages through the internalization and degradation of ferroportin, thereby regulating iron metabolism in various diseases, including CKD^41^. Serum hepcidin is reported to correlate positively with serum ferritin and negatively with the eGFR in patients with CKD^42^. Consequently, serum hepcidin may mediate the development of functional ID in CKD^43^. Although functional ID in previous large cohorts of CKD was reported to be associated with all-cause mortality^16,17^, whether this functional ID affects kidney health remains unclear^44^. Fourth, we did not examine the presence or absence of oral iron supplementation or dietary iron intake, and neither the duration of oral iron use nor the history of intravenous iron administration was evaluated before enrollment. Therefore, the present study did not reveal the precise iron load in each participant at enrollment. In the CRIC Study, the prevalence of patients’ self-reported oral iron use, including iron supplementation, by a dietary questionnaire was 36%^15^. In our cohort, the prevalence of oral iron use in our cohort may have been underestimated given the lack of inclusion of oral iron supplementation; nevertheless, the prevalence (5%) of its use was obviously lower than that in the CRIC Study. The median values of TSAT and serum ferritin in the CRIC Study were 22% and 158 ng/mL, respectively^15^, and those in our cohort were 32.8% and 139.2 ng/mL, respectively (Table 1). Despite the much lower oral iron use in our cohort than in the CRIC Study cohort, there was no large difference in the values of iron status markers between the two cohorts. In the CRIC Study, most participants were non-Hispanic white patients or non-Hispanic Black/African-American patients^45^. In a study of the general population, East Asian (Japanese, Chinese, Vietnamese, and Filipino) individuals showed significantly higher levels of iron status markers, such as TSAT and serum ferritin, than European, African-American, or South Asian individuals, and this difference was also significant for both males and females^46^. In another study, Asian woman exhibited significantly higher iron absorption than a group of age-matched Caucasian women^47^. With respect to the possible mechanisms explaining these ethnic differences, the storage threshold at which East Asians downregulate iron absorption was suggested to be higher^46^. Therefore, this higher iron storage threshold observed in East Asians may have contributed to the lack of a large difference in iron status markers between the CRIC Study cohort and our Japanese cohort, despite the large difference in the prevalence of oral iron use between the two cohorts. However, no studies to date have investigated the ethnic differences in iron status markers among patients with CKD. Thus, further studies are needed to determine whether such ethnic differences exist. Additionally, we did not evaluate the fluctuation in the iron status markers or oral iron use during the follow-up period. Finally, there was a high inter-method variability for serum ferritin, probably due to the wide variety of assay procedures, whereas inter-method variability was small for TSAT. Serum ferritin values were considered suited only for intra-hospital comparison^3,48^. Additionally, reference ranges of “normal” serum ferritin levels can vary, and the “normal” range is both assay- and laboratory-dependent^49^. Accordingly, reported serum ferritin levels should be interpreted with caution.

In conclusion, the present study demonstrated that lower and higher serum ferritin levels were associated with poor kidney outcome independent of relevant confounding factors. This J-shaped association may provide a novel insight into the influence of serum ferritin levels on kidney disease progression in patients with CKD not receiving dialysis.

## Methods

### Study design

Between September 2009 and July 2022, 1,217 consecutive Japanese patients were admitted to our nephrology division of our hospital, the NHO Kyushu Medical Center, for the evaluation of and education regarding CKD. Participants who were referred to our nephrology outpatient department from nearby hospitals or clinics or from other outpatient departments of our hospital for the evaluation of kidney dysfunction, urinary abnormalities, and/or abnormal kidney morphologies were admitted to our nephrology division for the evaluation of and education regarding CKD. Patients who had been followed up for CKD at our nephrology outpatient department and exhibited CKD-related problems such as poor blood pressure control, exacerbation of edema, progression of kidney disease, urinary abnormalities necessitating kidney biopsy, or polycystic kidney disease necessitating tolvaptan *w*ere also admitted to our division for further evaluations including adjustment of antihypertensive agents or diuretics, kidney biopsy, or initiation of tolvaptan. Among these patients, we excluded 102 who had a malignancy, 52 who exhibited acute-on-chronic kidney injury, 60 who had an eGFR < 8 mL/min/1.73 m^2^, 21 who received blood transfusion during hospitalization, 12 who had hematological disorders (e.g., anaplastic anemia, myelodysplastic syndrome, hemochromatosis, and a history of leukemia, multiple myeloma, or malignant lymphoma), 33 who lacked available data on blood samples, and 5 with serum ferritin > 1,000 ng/mL. The remaining 932 patients were discharged from our hospital without initiating kidney replacement therapy, and they were prospectively followed-up. Among these 932 patients, 42 were lost to follow-up within 6 months of discharge, and they were also excluded. Ultimately, data from 890 patients were prospectively analyzed. Data were collected until June 2023. After discharge, the participants were prospectively followed up every 1 to 6 months at our nephrology outpatient department. We checked each patient’s laboratory data at every outpatient visit and simultaneously evaluated the presence or absence of cardiovascular events or death in the medical record. For participants who were lost to follow-up, we irregularly checked the laboratory data, cardiovascular events, and presence or absence of death in each participant by inquiring in writing to other hospitals or clinics that they had visited. The latest surveillance was performed between March 2023 and June 2023. Based on these surveillances, we evaluated the time of doubling of the serum creatinine concentration, ESKD, or death from kidney disease and other causes. This study was approved by the Ethics Committee at the NHO Kyushu Medical Center (Approval Number: 09–09), was registered with the University Hospital Medical Information Network (UMIN000017519), and was performed in accordance with the guidelines of the Declaration of Helsinki. Written informed consent was obtained from all participants included in the study.

### Outcome definitions

The primary kidney endpoint was a composite of doubling of serum creatinine, ESKD, or death due to kidney failure. ESKD was defined as kidney dysfunction that required maintenance hemodialysis or peritoneal dialysis or that necessitated kidney transplantation. In this study, the follow-up period was defined as the time to the first event in patients who experienced kidney events and as the time to completion or loss to follow-up in patients with censoring. Participants who were lost to follow-up after being followed up for at least 6 months were included in the analysis as censored subjects.

### Data collection

Blood samples were obtained from each patient early in the morning following an overnight fast. Daily proteinuria was also measured. Serum ferritin was determined by an electrochemiluminescence immunoassay (Roche diagnostics Co., Ltd., Tokyo, Japan) (reference range: 39.9–465 ng/mL for men and 6.23–138 mg/mL for women). The eGFR (mL/min/1.73 m^2^) was calculated using the following new Japanese equation: eGFR = 194 × SCr^−1.094^ × age^−0.287^ × 0.739 (if female) where SCr is serum creatinine^50^. CKD was defined as the persistence of either of the following two conditions for more than 3 months: (1) Findings suggesting kidney damage; i.e., abnormal findings in blood or urinary tests, imaging studies, or pathological evaluations as well as evidence of proteinuria (≥ 0.15 g/g creatinine) and (2) GFR of < 60 mL/min/1.73 m^2^^51^. In accordance with the guideline of the Japanese Society for Dialysis Therapy, anemia was defined as follows: for male patients, hemoglobin < 13.5 g/dL (< 60 years), hemoglobin < 12.0 g/dL (60–69 years), and hemoglobin < 11.0 g/dL (≥ 70 years); for female patients, hemoglobin < 11.5 g/dL (< 60 years), hemoglobin < 10.5 g/dL (60–69 years), and hemoglobin < 10.5 g/dL (≥ 70 years)^38^.

All participants were carefully interviewed about their medical history, including hypertension, diabetes mellitus, prior CVD, and other comorbidities, including inflammatory disease. For each patient, demographic information (age and sex), medication history, and history of smoking at presentation were recorded. Patients requiring inpatient medical management for acute illnesses (e.g., severe infection, acute cardiac failure, and acute respiratory failure) besides CKD were not included in this cohort. Prior CVD was defined as a history of ischemic heart disease, heart failure, stroke, peripheral artery disease, and/or aortic dissection. Oral medications including oral iron, and use of erythropoiesis-stimulating agents were checked based on each patient’s medicine notebook and/or medical record. However, neither supplemental nor dietary iron intake was examined, and neither the duration of oral iron use nor the presence or absence of intravenous iron administration before admission was evaluated. Cigarette smoking was evaluated as current or past. The BMI was calculated as weight in kg divided by height in m^2^. Blood pressure was measured three separate times on day 2 of hospitalization with the patients in a sitting position. The mean of the three readings was recorded.

### Statistical analyses

Continuous data are expressed as the median (interquartile range) because the Shapiro–Wilk test indicated that all continuous variables were non-normally distributed. Patients were divided into quartiles (Q1–Q4) on the basis of their TSAT or serum ferritin. Spearman’s rank correlation coefficients were used to identify the association between two continuous variables. A trend analysis was performed using the *nptrend* command for Stata (non-parametric test for trend). A Cox proportional hazard model was used to determine whether TSAT or serum ferritin levels were associated with kidney outcome. The HRs and 95% CIs were calculated for each variable. We selected traditional cardiovascular risk factors for kidney disease progression (age, smoking, diabetes mellitus, systolic blood pressure, dyslipidemia, and BMI), nontraditional cardiovascular risk factors for kidney disease progression (hemoglobin, C-reactive protein, daily proteinuria, serum albumin, serum phosphorus, and eGFR), prior CVDs, use of immunosuppressants and RAAS inhibitors that affect kidney outcome, use of erythropoiesis-stimulating agents and presence of chronic inflammatory disease that affect iron status, and serum ferritin (for TSAT) or TSAT (for serum ferritin) that is associated with each other as covariates. Death from any cause other than kidney disease before kidney events was considered to be a competing event^52^. Therefore, a Fine-Gray proportional subdistribution hazard model was also performed by taking into account the competing risk of an alternative outcome (all-cause death) on the association between iron status markers and kidney outcome. We evaluated the proportionality assumption by creating logarithmic hazard plots of kidney survival probability against the logarithm of analysis time. We also conducted a generalized linear regression considering the non-zero slope in relation to the function of time with the scaled Schoenfeld residuals. The log–log survivor functions by TSAT and serum ferritin levels were found to be parallel, and the regression line fit to the scaled Schoenfeld residuals did not show a non-zero slope (Supplementary Fig. 1). The proportional hazard assumption was thereby verified. We placed four knots at the 5th, 35th, 65th, and 95th percentiles of TSAT and serum ferritin levels, and a median TSAT value (36.5%) in the reference group (Q3) and a median serum ferritin value (102.9 ng/mL) in the reference group (Q2) were selected as a reference for spline plots. Subgroup analyses were performed according to sex, the presence or absence of categorical variables, and the status of continuous variables (below and above the median). Interaction effects of quartiles of serum ferritin and other variables on kidney outcome were estimated by adding interaction terms between quartiles of serum ferritin and other variables to the relevant model. All statistical analyses were performed using STATA version 14 (Stata Corp., College Station, TX, USA), with *P* < 0.05 indicating statistical significance.

### Supplementary Information

Supplementary Figure 1.

## Data Availability

The datasets generated during and/or analyzed during the current study are available from the corresponding author on reasonable request.
