# Supplementary material for: Association between iron status markers and kidney outcome in patients with chronic kidney disease
Source: Sci Rep. 2023 Oct 25;13:18278. doi: 10.1038/s41598-023-45580-8 (PMC10600187; doi:10.1038/s41598-023-45580-8)
Supplement: Supplementary file 1 — Supplementary Figure 1. [file 41598_2023_45580_MOESM1_ESM.pptx]

## Slide 1
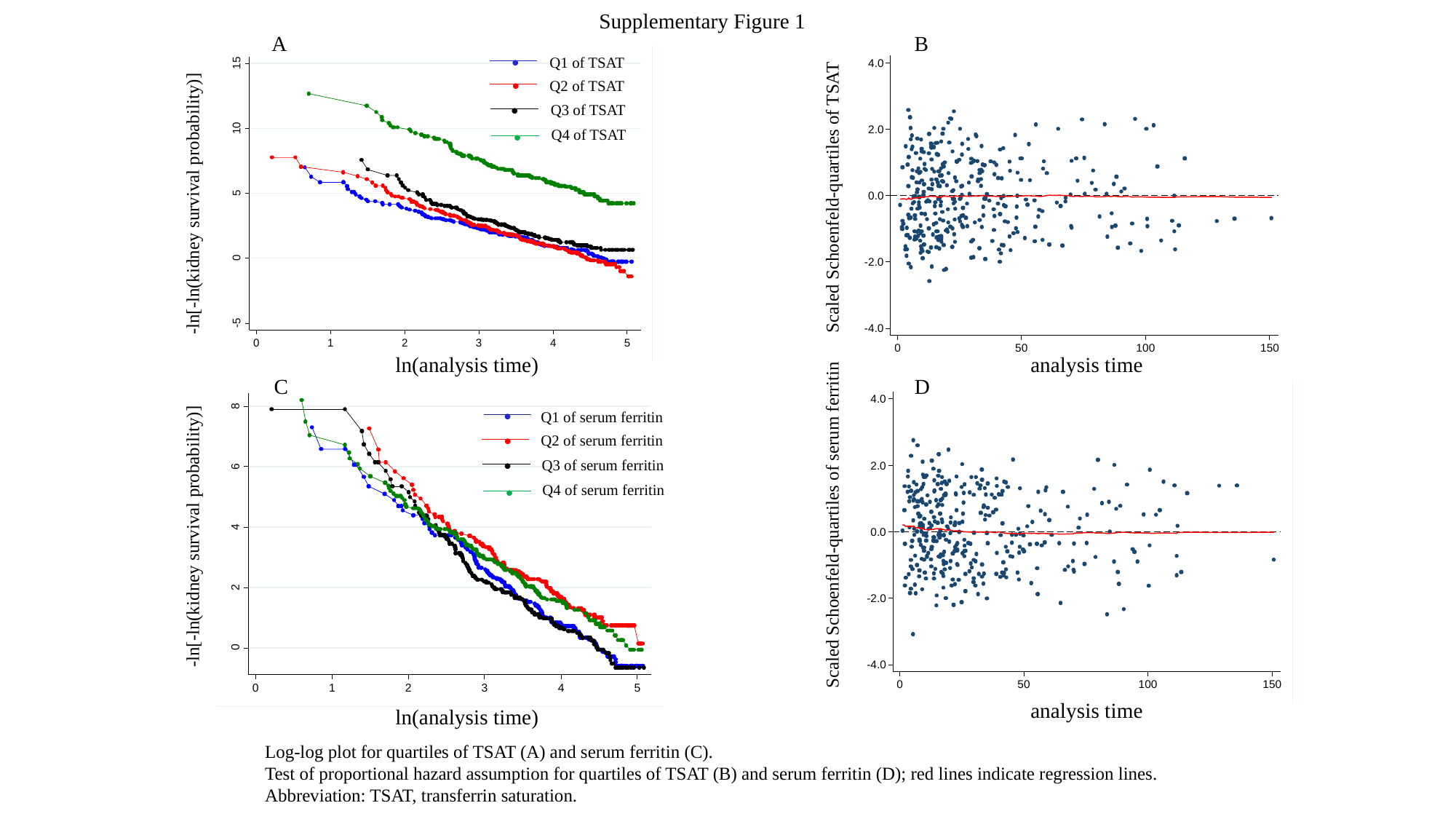

Supplementary Figure 1
A
B
Q1 of TSAT
●
Q2 of TSAT
●
Q3 of TSAT
●
Q4 of TSAT
●
-ln[-ln(kidney survival probability)]
Scaled Schoenfeld-quartiles of TSAT
analysis time
ln(analysis time)
C
D
Q1 of serum ferritin
●
Q2 of serum ferritin
●
Q3 of serum ferritin
●
Q4 of serum ferritin
●
-ln[-ln(kidney survival probability)]
Scaled Schoenfeld-quartiles of serum ferritin
analysis time
ln(analysis time)
Log-log plot for quartiles of TSAT (A) and serum ferritin (C).
Test of proportional hazard assumption for quartiles of TSAT (B) and serum ferritin (D); red lines indicate regression lines.
Abbreviation: TSAT, transferrin saturation.
